# TGFβ/cyclin D1/Smad-mediated inhibition of BMP4 promotes breast cancer stem cell self-renewal activity

**DOI:** 10.1038/s41389-021-00310-5

**Published:** 2021-03-01

**Authors:** Gang Yan, Meiou Dai, Chenjing Zhang, Sophie Poulet, Alaa Moamer, Ni Wang, Julien Boudreault, Suhad Ali, Jean-Jacques Lebrun

**Affiliations:** 1 McGill University Health Center, Department of Medicine, Cancer Research Program, Montreal, QC H4A 3J1 Canada; 2 Department of Gastroenterology, Zhejiang Provincial People’s Hospital, People’s Hospital of Hangzhou Medical College, Hangzhou, Zhejiang China

**Keywords:** Breast cancer, Cancer stem cells

## Abstract

Basal-like triple-negative breast cancers (TNBCs) display poor prognosis, have a high risk of tumor recurrence, and exhibit high resistance to drug treatments. The TNBC aggressive features are largely due to the high proportion of cancer stem cells present within these tumors. In this study, we investigated the interplay and networking pathways occurring between TGFβ family ligands in regulating stemness in TNBCs. We found that TGFβ stimulation of TNBCs resulted in enhanced tumorsphere formation efficiency and an increased proportion of the highly tumorigenic CD44^high^/CD24^low^ cancer stem cell population. Analysis of the TGFβ transcriptome in TNBC cells revealed bone morphogenetic protein4 (BMP4) as a main TGFβ-repressed target in these tumor cells. We further found that BMP4 opposed TGFβ effects on stemness and potently decreased cancer stem cell numbers, thereby acting as a differentiation factor in TNBC. At the molecular level, we found that TGFβ inhibition of BMP4 gene expression is mediated through the Smad pathway and cyclin D1. In addition, we also found BMP4 to act as a pro-differentiation factor in normal mammary epithelial cells and promote mammary acinar formation in 3D cell culture assays. Finally, and consistent with our in vitro results, in silico patient data analysis defined BMP4 as a potential valuable prognosis marker for TNBC patients.

## Introduction

Triple-negative breast cancers (TNBCs) represent 10–20% of all breast cancers and are characterized by negative or low estrogen receptor (ER), progesterone receptor (PR), and human epidermal growth factor receptor 2 (HER2) expression^1^. Based on their gene expression profiles, the majority of TNBCs are classified as basal-like breast cancers. This molecular subtype is often associated with larger tumor size, higher tumor grade, greater lymph node spread, and a higher rate of distant metastasis^2,3^. Classification based on gene expression analyses revealed that TNBC can be categorized into six subgroups, including basal-like (BL1 and BL2), mesenchymal (M), mesenchymal stem-like (MSL), immunomodulatory (IM), and luminal androgen receptor (LAR)^1^. The basal-like (BL1 and BL2) subtypes are highly enriched in gene expression patterns associated with proliferation and DNA damage-related genes while the mesenchymal (M and MSL) subtype shows high expression of epithelial-to-mesenchymal transition-related genes^1,4^. The immunomodulatory subtype presents gene ontologies for immune cell processes, including cytokine signaling as well as antigen processing and presentation^5,6^. Finally, the LAR subtype shows enrichment in genes related to the androgen receptor (AR) signaling and has been associated with a better prognosis compared to other TNBC subtypes^7,8^. Despite initial response to adjuvant chemotherapy, TNBC patients typically develop distant recurrence within 5 years of diagnosis^3^. Due to the molecular heterogeneity of TNBC and the absence of well-defined molecular targets, efficacious treatments for TNBC patients remain largely unavailable. Cancer stem cells (CSCs) or tumor-initiating cells represent a distinct subpopulation of cancer cells within the tumor, that possess stem cell-like properties^9^. These cells exhibit a long-term, self-renewal capacity, and can divide through asymmetric division, thereby continuously regenerating and propagating the heterogenous tumor^10^. CSCs have been implicated in tumor growth and progression, drug resistance, as well as in cancer recurrence^11^. Breast cancer stem cells (BCSCs) were initially identified as a small subpopulation of patient-derived breast cancer cells expressing CD44^+^/CD24^−/low^ cell surface markers^12^. Tumor-derived CD44^+^/CD24^−/low^ cells are able to form tumorspheres in vitro when cultured under anchorage-independent conditions in serum-free medium^13^. In contrast, cells that do not express these markers do not generate tumorspheres and have lower tumorigenic potential^14^. BCSCs are frequently detectable in metastatic pleural effusions of breast cancer patients or early-disseminated cancer cells in the bone marrow and are resistant to chemotherapy treatment in breast cancer patients^15,16^. Of note, tumor cells derived from basal-like or triple-negative breast cancers are enriched in CD44^+^/CD24^−/low^ subpopulations^17^. Thus, the stem cell-like properties of BCSCs may account for the poor prognosis, high tumor recurrence, and chemotherapy resistance in TNBC patients.

The TGFβ superfamily of growth factors includes over 30 members that can be categorized under the TGFβ/Activin, bone morphogenetic protein (BMP), and distant member’s main subgroups^18^. All members of the TGFβ superfamily exert pleiotropic effects throughout the body^18^. TGFβ itself, the founding member of this family plays an important role in regulating BCSCs^19–23^. Human mammary epithelial cells undergoing an epithelial-to-mesenchymal transition in response to TGFβ and Wnt signaling have been shown to acquire stem cell-like features^24^. Moreover, TGFβ signaling is specifically activated in CD44^+^/CD24^−/low^ BCSCs, leading to a mesenchymal and migratory phenotype^25^. It was also shown that TGFβ-induced tumorsphere formation occurs predominantly in claudin^low^ breast cancer (also known as basal-b subtype), as opposed to other breast cancer molecular subtypes^26^. Despite the accumulating evidence for the role of TGFβ in the regulation of BCSC function, the downstream targets and signaling pathways that mediate the TGFβ effects remain to be fully understood. BMP4, another member of the TGFβ superfamily plays fundamental roles in osteogenesis but also acts as a multipotent stem cell differentiating factor^27^. BMP4 has been shown to exert antitumor effects and to be able to re-sensitize tumors to therapy by differentiating stem-like cells in a glioma^28^.

The cell cycle regulator, cyclin D1, can promote stem cell expansion and inhibit differentiation of several embryonic, hematopoietic, and normal mammary progenitor cells^29,30^. Cyclin D1 also plays an important role during mammary gland development, as cyclin D1-knockout mice fail to generate lobuloalveoli in the mammary glands during pregnancy^31^. Interestingly, cyclin D1 is frequently overexpressed in human breast, melanoma, prostate, lung, and oral squamous cell carcinomas^32–34^. Moreover, elevated cyclin D1 expression associates with a high incidence of tumor metastasis and poor survival outcome^35^, and its overexpression has been shown to promote the initiation and development of breast cancer^36^. We have previously shown that cyclin D1 acts downstream of TGFβ to regulate breast cancer cell migration and invasion, two key features of CSC activity^37^. Moreover, our lab recently found that the cyclin D1 associated kinase, CDK4 can regulate cancer stemness in TNBC^38^. We thus, hypothesized that cyclin D1 may also regulate BCSC self-renewal activity, downstream of TGFβ.

In this study, we show that TGFβ promotes stemness and negatively regulates BMP4 expression in TNBC through the canonical Smad pathway and cyclin D1. We further found cyclin D1 to be highly expressed in tumorspheres compared to cells in monolayer cultures, consistent with a role in promoting stemness. Conversely, we show that BMP4 potently inhibited tumorsphere formation and reduced CD44^+^/CD24^−/low^ numbers in BC cells. Interestingly, BMP4 also promoted differentiation of normal mammary epithelial cells, highlighting BMP4 as a potent pro-differentiation factor in both normal and breast cancer cells. Together these results define an antagonistic feedback loop and signaling network between TGFβ superfamily members, whereby TGFβ/Smad/cyclin D1 signaling leads to increased cancer stem cell numbers while BMP4 oppose these effects acting as a potent differentiation factor.

## Methods

### Cell lines

All TNBC SUM cell lines were obtained from Stephen Ethier (The Medical University of South Carolina). The SCP2 cell line was kindly provided by Dr. Joan Massagué (Sloan Kettering Institute). All the cell lines were routinely tested by Diagnostic Laboratory from Comparative Medicine and Animal Resources Centre (McGill University).

### Cell culture

Human breast cancer cell line SUM159PT, SUM149PT, and SUM229PE were cultured in Ham’s F-12 nutrient mixture (Sigma-Aldrich) supplemented with 5% fetal bovine serum (FBS), 5 µg/ml insulin, and 1 µg/ml hydrocortisone. Human breast cancer cell line SCP2 was cultured in DMEM (Sigma-Aldrich) containing 10% FBS and 2 mM L-glutamine. For cell transfection, please refer to Supplementary Materials and Methods.

### Tumorsphere formation and flow cytometry assays

SUM159PT cells were seeded at 10,000 cells per well in 12-well low-attachment plates and grown for 5–7 days in Ham’s F-12 nutrient mixture supplemented with B27, 10 ng/ml EGF, and 10 ng/ml bFGF. For detailed tumorsphere scoring and flow cytometry analysis, please refer to Supplementary Materials and Methods.

### Real-time PCR

SUM159PT, SUM149PT, SUM229PE, and SCP2 cells were lysed by TRIzol reagent (Invitrogen), and the total RNA was extracted following the standard procedures. For detailed reverse transcription and PCR steps, please refer to Supplementary Materials and Methods.

### Western blot analysis

Antibodies and reagents were obtained from Thermo Scientific and Santa-Cruz. For detailed information, please refer to Supplementary Materials and Methods.

### Luciferase assay

The series of 5′-progressive deletion of the human BMP4 gene promoter fused to the luciferase gene (3.36kb-BMP4-luc, 3.17kb-BMP4-luc, 2.10kb-BMP4-luc, 1.7kb-BMP4-luc, and 0.46kb-BMP4-luc) were kindly provided by Dr. Daniel Chung^39^. For complete steps, please refer to Supplementary Materials and Methods.

### 3D cell culture

The morphology of mammary epithelial organoids was evaluated after 72 h of different treatments. For complete steps, please refer to Supplementary Materials and Methods.

### Immunofluorescence staining and confocal microscopy

mammary organoids in 3D culture were fixed in 4% PFA and permeabilized in 0.5% Triton X-100/1XPBS (PBST) before immunostaining. For complete procedures, please refer to Supplementary Materials and Methods.

### Gene expression profiling

SCP2 cells were serum-starved overnight and treated with 100 pM TGFβ1 for 24 h in a serum-free medium. Total RNA samples were extracted using the TRIzol reagent (Invitrogen). For complete steps, please refer to Supplementary Materials and Methods.

### Online data analysis

GOBO, TCGA-BRCA datasets were used to assess BMP4 expression levels in different breast molecular subtypes. The GOBO database was further applied to analyze BMP4 expression levels according to the ER status and tumor grade. The patient numbers in each category are indicated in the corresponding figures. Kaplan–Meier plotter was used to evaluate the association between BMP4 and TGFβ mRNA level and clinical outcome represented as relapse-free survival (RFS).

### Statistical analyses

All results are presented as the mean ± SEM for at least three repeated individual experiments. The difference between groups was analyzed using Student’s *t* test, and *P ≤ 0.05 was considered statistically significant.

## Results

### TGFβ transcriptomic analysis in TNBC cells

**Fig. 1:**
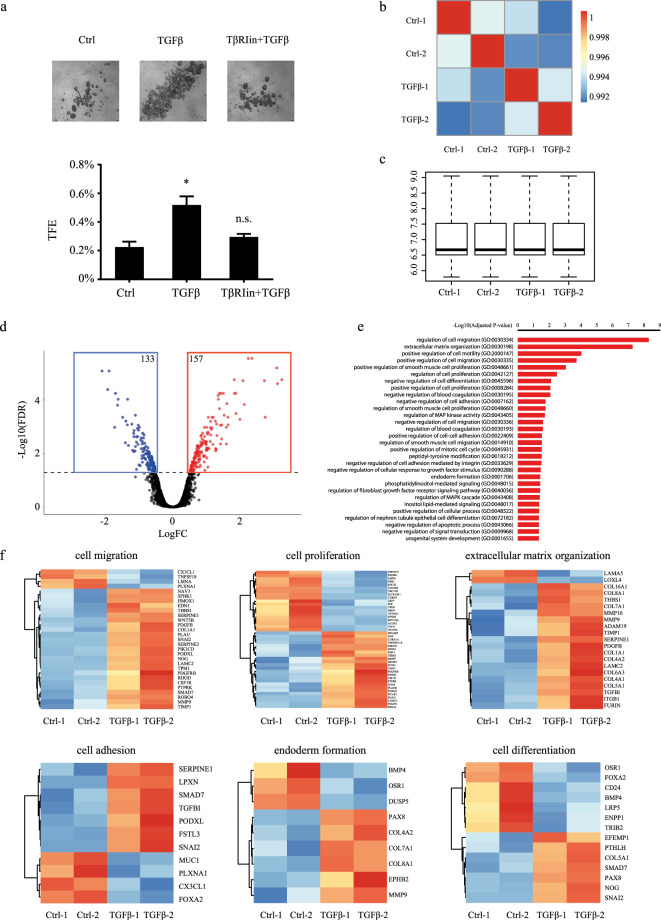
TGFβ transcriptomic analysis in TNBC cells. **a** TGFβ effects on tumorsphere formation. Data are expressed as mean ± standard error. *P ≤ 0.05, n.s. not significant. **b** Pearson correlations and (**c**) normalized counts across all bioreplicates. **d** Volcano plot of differential expressed genes (red and blue indicate up- and downregulated genes, respectively (FDR < 0.05). **e** Gene ontology enrichment analysis of 290 candidate genes (FDR < 0.05) using EnrichR. **f** Heatmaps of the TGFβ-regulated biological processes.

To start analyzing the TGFβ role on BCSC biology in TNBC, we first examined the TGFβ effects on tumorsphere formation. In this type of assay, cancer stem/progenitor cells are enriched in serum-free, nonadherent culture conditions, allowing for proper identification and quantitation of cancer stem cell numbers. We used TNBC SUM159PT cells, a TNBC cell line derived from a patient with anaplastic carcinoma^40^. SUM159PT cells were seeded at moderate seeding density (10,000 cells) in the presence or the absence of TGFβ (100 pM), under low-attachment culture conditions, as described in “Methods”. Tumorsphere forming efficiency (TFE) was determined as the number of tumorspheres divided by the number of single cells seeded, expressed as a percentage. As shown in Fig. 1a, TFE tumorsphere numbers were significantly increased in cells treated with TGFβ compared to control. This effect is mediated through the classical TGFβ receptor signaling pathway, as the addition of a specific TGFβ receptor I kinase inhibitor (TβRIin) significantly blocked TGFβ-induced tumorsphere formation (Fig. 1a). These data indicate that activation of the TGFβ signaling pathway promotes BCSC activity and self-renewal in TNBC.

**Fig. 2:**
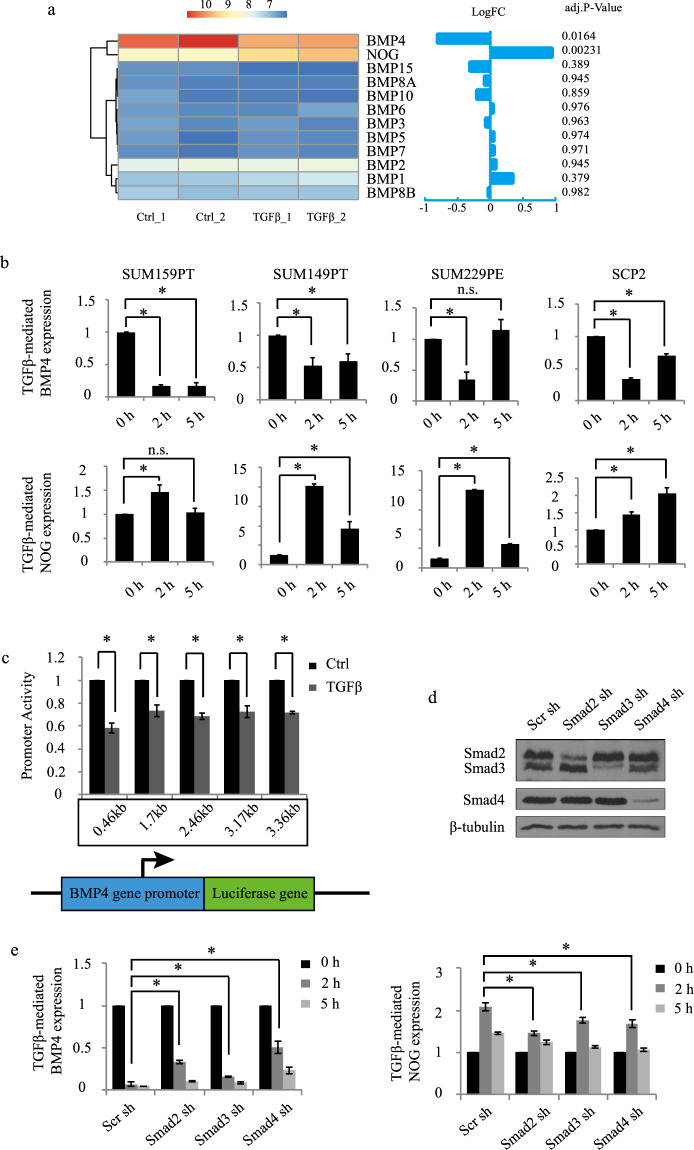
BMP4 and NOG are inversely regulated by TGFβ. **a** Heatmap representing the TGFβ effects on BMP family members and NOG expression with log fold change and adjusted *P* value. **b** QPCR analysis of BMP4 and NOG in various TNBC cell lines. Data represent means ± SEM of triplicate experiments. *P ≤ 0.05; n.s. not significant. **c** TGFβ effects on progressive deletion constructs of the BMP4 gene promoter fused to luciferase. Data were normalized to the control group, and graphs are means ± SEM from triplicate data. *P ≤ 0.05; n.s. not significant. **d** Immunoblots of the Smad knockdown efficiencies. **e** Smad knockdown effects on TGFβ-mediated BMP4 and NOG expression.

To further address the molecular mechanisms by which TGFβ regulates tumor initiation in TNBC, we performed a microarray analysis, using the Illumina Human HT-12 Gene Expression BeadChip in TNBC cells treated or not with TGFβ for 24 h. The high screen efficiency and sample correlation were reflected by the high Pearson correlation coefficient (>0.99) (Fig. 1b) and overall consistent signal intensity across biological replicates (Fig. 1c). As shown in Fig. 1d, differential gene expression (DGE) analysis using a threshold cutoff (FDR < 0.05) revealed 290 TGFβ-regulated downstream target genes, with 157 upregulated and 133 downregulated targets. A gene ontology enrichment analysis (GOEA) was then performed using EnrichR^41,42^ (https://amp.pharm.mssm.edu/Enrichr/) and highlighted cell migration, extracellular matrix organization, cell motility cell proliferation, and cell differentiation as top-ranking biological functions among the 290 identified targets (Fig. 2e). Collapsing biological process (BP) terms based on functional similarity allowed for the visualization of various gene expression profiles specific to each biological function (Fig. 2f). These results are consistent with the well-described effects of TGFβ signaling on cell migration, motility, invasion, and proliferation in cancer cells^18,43^, further demonstrating the stringency and relevance of our microarray analysis. Interestingly, besides the hallmark TGFβ effects, negative regulation of cell differentiation also came out as a top-ranking biological function for the 290 identified TGFβ target genes. This is consistent with our data showing TGFβ as a potent stemness factor in TNBC (Fig. 1a) and suggested that TGFβ may exert its antidifferentiation effects through downregulation of cell differentiation genes. In particular, we found TGFβ to potently downregulate the expression of BMP4, a known cell differentiation factor, while upregulated the BMP4 antagonist Noggin (Fig. 1f). BMP4 is also a member of the TGFβ superfamily, thus suggesting the existence of a negative feedback loop between TGFβ family members to regulate the balance between cancer stemness and differentiation.

### TGFβ inhibits BMP4 gene expression

As described in “Introduction”, BMP4 plays a role as a differentiation factor in glioma^28^. We thus hypothesize that TGFβ could promote BCSC numbers and stemness through inhibition of BMP4 signaling in TNBC. Using our microarray data, we first investigated the specificity of the TGFβ effects on all BMP family member’s gene expression in TNBC and found that TGFβ only regulates BMP4 expression and that no other BMP family members were significantly regulated by TGFβ (Fig. 2a). Interestingly, our transcriptomic analysis also revealed that TGFβ could significantly upregulate the expression of the BMP4 inhibitor, Noggin (NOG). To avoid the limitation of the use of a single-cell line, we then examined the TGFβ effects on BMP4 and NOG expression in a panel of human triple-negative breast cancer cell lines (SUM159PT, SUM149PT, SUM229PE, SCP2). SUM159PT is derived from an anaplastic carcinoma with mesenchymal phenotype; SUM149PT is derived from an invasive ductal carcinoma, inflammatory histotype, with Basal B phenotype; and SUM229PE is derived from a pleural effusion related to breast cancer with Basal B phenotype. The SCP2 cell line is a single-cell-derived progeny (SCP) derived from the in vivo selection of bone-specific metastatic cells from the human breast cancer TNBC cell line MDA-MB-231^44^. SCP2 cells are capable of bone metastasis and pre-exist within the MDA-MB-231 parent line, which was originally established as the total outgrowth of cells derived from a pleural effusion of a patient who relapsed years after removal of the primary tumor^45^. We found that TGFβ could potently inhibit BMP4 expression while increasing Noggin in all cell lines tested, as early as 2 h following stimulation of the cells (Fig. 2b). This effect appears to be mediated at the transcriptional levels, as TGFβ could significantly repress the activity of a series of progressive BMP4 gene promoter deletion constructs fused to luciferase reporter constructs (Fig. 2c). TGFβ efficiently inhibited the activity of the shortest promoter construct (460 bp) further indicating that the TGFβ regulatory sequences are located within the proximal region of the BMP4 gene promoter, close to the 5′ transcription initiation start site.

TGFβ classically regulates the expression of its target genes through the canonical Smad pathway, through Smad2, 3, and 4^18^. To then assess whether the TGFβ effects on BMP4 and NOG expression were Smad-dependent, TNBC (SUM159PT) cells were transfected with specific shRNAs targeting Smad2, 3, or 4 or a scrambled shRNA as a negative control. As shown in Fig. 2d, the efficacy and specificity of each shRNA were assessed by immunoblotting using specific antibodies against the Smads. Effects of the Smad knockdowns on BMP4 and Noggin expression were then assessed and quantified by qPCR and revealed that all Smad individual knockdowns significantly blocked TGFβ-mediated inhibition of BMP4 expression and TGFβ-induced NOG expression (Fig. 2e). Together, these results that TGFβ/Smad signaling strongly antagonizes BMP4 signaling through multiple pathways, including direct repression of BMP4 gene expression with concomitant up-regulation of the BMP4 inhibitor, Noggin.

### Cyclin D1 is a downstream mediator of TGFβ-induced BMP4 downregulation

**Fig. 3:**
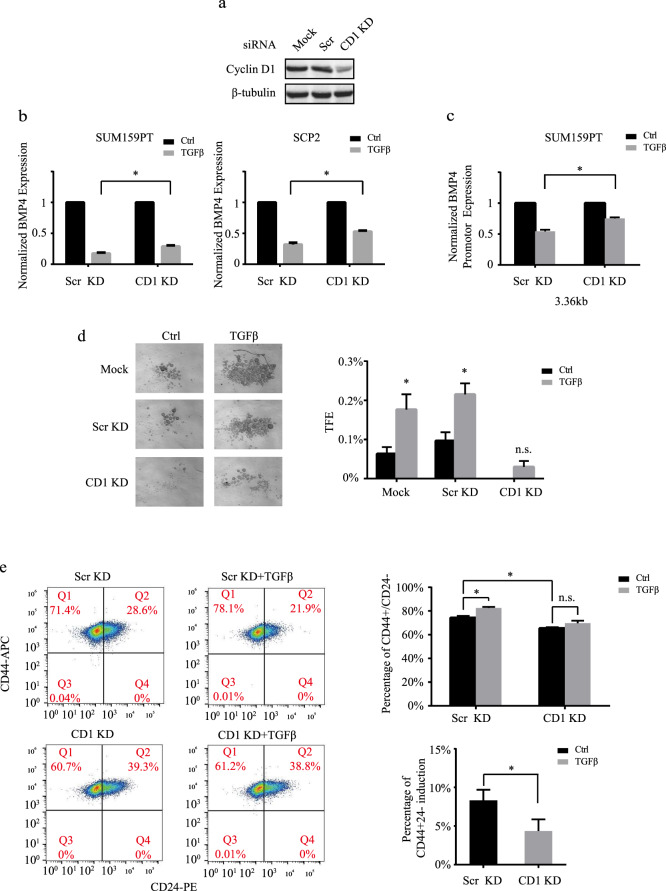
Cyclin D1 is required for TGFβ inhibition of BMP4. **a** Immunoblot analysis to assess cyclin D1 knockdown efficiency. **b** Cyclin D1 knockdown effects on TGFβ-mediated BMP4 expression. **c** Cyclin D1 knockdown effects on TGFβ-mediated BMP4 gene promoter inhibition. **d**, **e** Cyclin D1 knockdown effects on TGFβ-mediated tumorsphere formation (**d**) and TGFβ-induced CD44^high^/CD24^−/low^ cell numbers (**e**). Data represent means ± SEM of triplicate experiments. *P ≤ 0.05; n.s. not significant.

We previously identified cyclin D1 as an important player downstream of TGFβ signaling in TNBC and showed that TGFβ itself could upregulate cyclin D1 expression^37^. Besides acting as a cell cycle regulator, cyclin D1 was also shown to act as an important proto-oncogene. In fact, cyclin D1 is frequently deregulated in multiple tumor types and overexpressed through copy number variation in over 50% of breast cancer patients^46^. To then address whether TGFβ-mediated regulation of BMP4 and stemness also involves cyclin D1 in TNBC, we knockdown cyclin D1 expression by means of RNA interference (Fig. 3a). Interestingly, as shown in Fig. 3b, the TGFβ-mediated inhibition of BMP4 gene expression was strongly impaired in the absence or reduced levels of cyclin D1. Similarly, when cyclin D1 was knockdown, the TGFβ inhibitory effects on BMP4 gene promoter activity were significantly reversed (Fig. 3c), indicating that TGFβ-mediated regulation of BMP4 requires cyclin D1. Having shown that TGFβ inhibits BMP4 while promotes stemness, we next assessed the role and contribution of cyclin D1 in controlling cancer stem cell numbers. The two main CSC populations present in breast cancer are of epithelial stem-like (ADLH+) and mesenchymal stem cell-like phenotype (CD44^high^/CD24^low^) origins. Importantly, while ADLH+ CSCs are enriched in the HER2+ subtype, they only represent a minority CSC population in TNBC. Indeed, the most prominent CSC population in TNBCs are the mesenchymal CD44^high^/CD24^low^ cancer stem cells, which are known to drive the aggressive nature of TNBC tumors. Thus, to start to investigate and characterize the TGFβ/BMP4 signaling cross-talk/network and stemness/pro-differentiation effects in TNBC, we examined these growth factor’s effects on tumorsphere formation (to reflect global CSC numbers) and specifically analyzed their effects on the predominant CD44^high^/CD24^low^ CSC subpopulation in those tumors. As shown in Fig. 3d, TGFβ strongly increased tumorsphere numbers in TNBC but these effects were significantly reduced in the absence of cyclin D1. As indicated above, a major CSC group in TNBC is represented by the CD44^high^/CD24^−/low^ cancer stem cell population. CD44^high^/CD24^−/low^ breast cancer cells display greater stem cell-like features and tumorigenic capacity compared to CD44^−^ and CD24^+^ cells^47^. We thus examined the TGFβ and cyclin D1 knockdown effects on this CSC population using flow cytometry, as we previously described^23,38^. As shown in Fig. 3e, while TGFβ significantly increased the CD44^high^/CD24^−/low^ cell numbers, this effect was blocked in the absence of cyclin D1. The flow cytometry results are in line with our tumorsphere assay data and further indicate the requirement of cyclin D1 for TGFβ to promote stemness in breast cancer.

### BMP4 acts as a differentiation factor and inhibits TGFβ-induced stemness

**Fig. 4:**
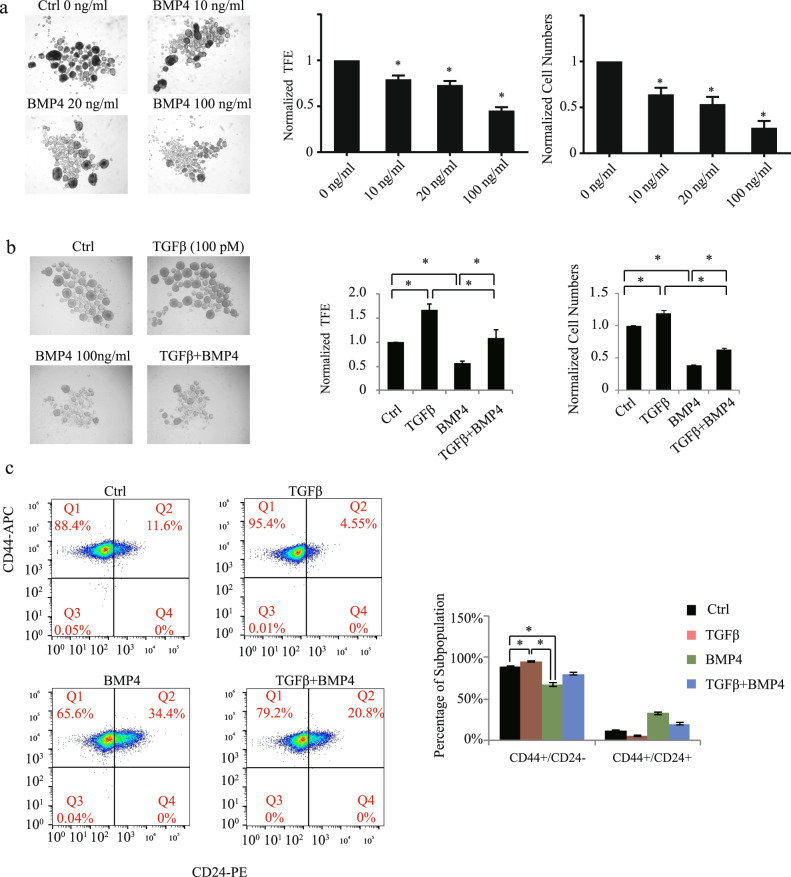
BMP4 acts as a differentiation factor and inhibits TGFβ-induced stemness. Tumorsphere formation assay showing that BMP4 inhibits basal (**a**) and TGFβ-induced (**b**) cancer stem cell activity. Data represent means ± SEM of triplicate experiments. *P ≤ 0.05. **c** Flow cytometry to assess TGFβ and BMP4 effects on CD44^high^/CD24^−/low^ CSC numbers. Data represent means ± SEM of triplicate experiments. *P ≤ 0.05.

We next sought to further characterize the BMP4 pro-differentiation role in TNBC and investigate the antagonistic effects played by TGFβ/BMP4 in the regulation of stemness in TNBC. For this, SUM159PT cells were treated or not with different concentrations of BMP4 for 7 days, as indicated in Fig. 4a before being assessed for tumorsphere efficiency and cell numbers (after tumorspheres were dissociated into single tumor cells). As shown in Fig. 4a, we found increasing BMP4 concentrations to concomitantly decrease tumorsphere efficiency and cell numbers for up to 50% and 75%, respectively, when using the highest BMP4 dose (100 ng/ml). Conversely, as shown in Fig. 4b, TGFβ could increase both tumorsphere efficiency and tumor cell numbers but these effects were antagonized and reversed when both TGFβ and BMP4 were added, suggesting that restoring BMP4 signaling and cell differentiation could block TGFβ-mediated stemness. Similarly, when assessing these growth factor effects on the CD44^+^/CD24^−/low^ cancer stem cell population, we found that BMP4 acted as a differentiation factor, able to decrease both basal and TGFβ-induced BCSC numbers (Fig. 4c). Altogether, these results indicate that the two family members, BMP4 and TGFβ, antagonize each other effect in the regulation of cancer stemness and highlight BMP4 as a potent pro-differentiation factor in TNBC.

### BMP4 differentiates mammary epithelial cells into an acinar structure in 3D cell culture

**Fig. 5:**
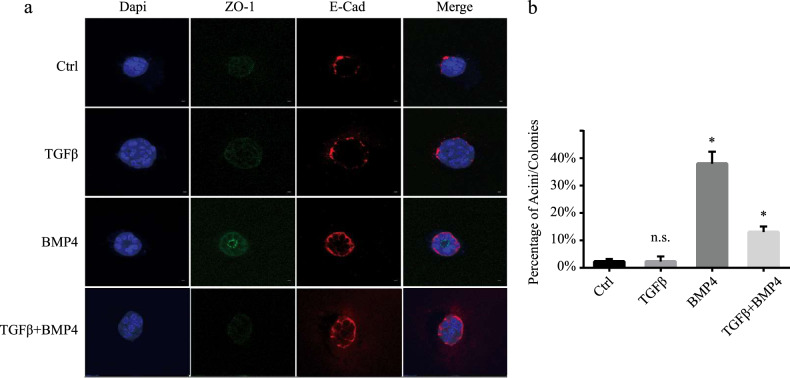
BMP4 induces mammary acinar structure in 3D cell culture. **a** 3D culture of mouse primary mammary epithelial cells stained with ZO-1 (green), E-cadherin (red), and Dapi (blue). **b** percentage of mammary acini total colonies (>100 colonies in triplicates). Graph shows mean ± SEM of triplicates of three independent experiments. *P ≤ 0.05; n.s. not significant.

We next evaluated whether BMP4 could act as differentiation and a polarity morphogenic factor in normal mammary epithelial cells to induce the formation of mammary acinar structures. For this, we performed ex vivo acini morphogenesis assays as described previously^48^ using primary mammary epithelial cells isolated from female virgin mice. As indicated in Fig. 5a, BMP4 stimulation strongly induced the formation of organized mammary acini with well-established apical/basal polarity as indicated by the apical localization of ZO-1 and basal/lateral localization of E-cadherin. On the other hand, control and TGFβ stimulated cells did not show any organized acini-like structures. Interestingly, stimulation of the cells with TGFβ, in addition to BMP4, strongly antagonized the BMP4 effects on acinar morphogenesis. Having shown the BMP4/TGFβ effects on acinar structures, lumen formation, and polarity, we then quantified the numbers of acini observed in the different conditions. As shown in Fig. 5b, the acinar formation efficiency (percentage of acini/colonies) was significantly increased by BMP4 treatment and this effect was antagonized in the presence of TGFβ. Together, these results highlight BMP4 as a potent differentiation factor in normal mammary epithelial cells, able to promote the formation of well-organized 3D acinar structures and show that TGFβ can efficiently antagonize these BMP4 differentiation effects.

### BMP4 expression correlates with least aggressive breast cancer subtypes and is associated with beneficial clinical features

**Fig. 6:**
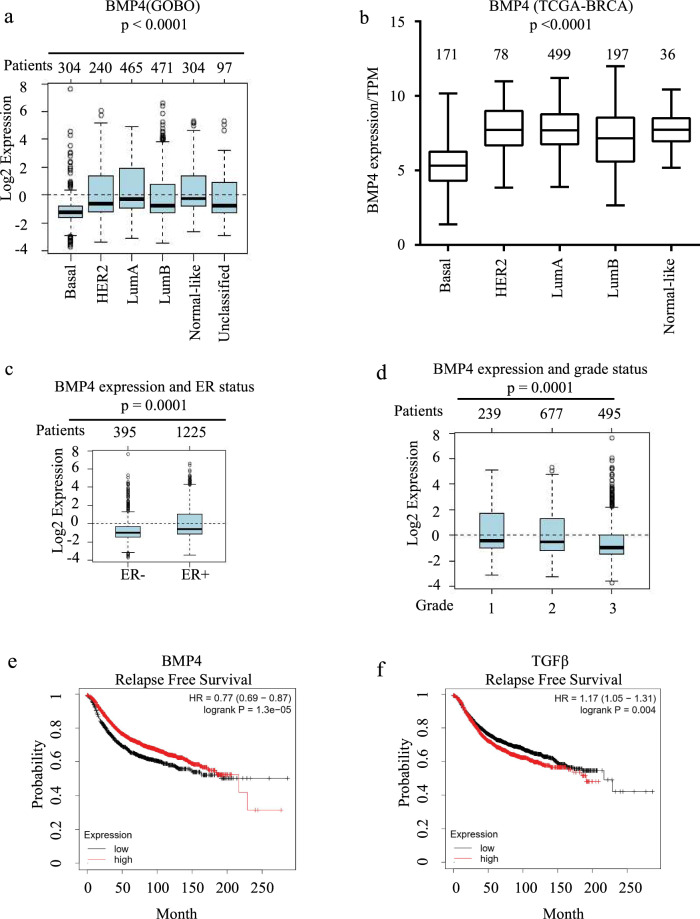
BMP4 expression correlates with least aggressive breast cancer subtypes and is associated with beneficial clinical features. **a**, **b** Boxplot of BMP4 expression across different breast cancer subtypes using GOBO (**a**) and TCGA-BRCA (**b**) datasets. The number of patients for each subtype is indicated. **c**, **d** Boxplot of BMP4 expression in breast cancer patients classified by ER status (**c**) and tumor grades (**d**). **e** Kaplan–Meier survival analysis for RFS by splitting patients into low and high BMP4 expression groups. **f** Kaplan–Meier relapse-free survival analysis for TGFβ.

Having shown that BMP4 acts as a differentiation factor in both normal and cancer cells, able to decrease BCSC numbers, we then investigated its potential as a predictive molecular marker for breast cancer patients. For this, we performed bioinformatics analysis using GOBO^49^ and TCGA-BRCA^50^ online databases to identify any correlation between BMP4 gene expression and breast cancer clinical features. We first analyzed BMP4 mRNA expression levels across different breast cancer molecular subtypes. As shown in Fig. 6a, analysis of the GOBO database revealed BMP4 expression levels to be the highest in the least aggressive luminal A subtype, while being the lowest in the most aggressive, invasive basal subtype. Analysis of the TCGA-BRCA dataset revealed a similar pattern (Fig. 6b), indicating that the lowest BMP4 expression levels correlate with the most aggressive breast cancer subtypes. Moreover, as shown in Fig. 6c, BMP4 expression was significantly higher in ER+ tumors compared to ER− tumors, consistent with the fact that cancer stem cell markers are usually associated with ER status and predictive of a poor survival outcome in ER− patients^51^. Tumor grade represents a clear indicator of the differentiation stage and growth rate of tumor cells. Whereas grade 1 tumors are well-differentiated with a slow growth index, grade 2 tumors are moderately differentiated with an intermediate growth index, while grade 3 tumors exhibit high CSC content and very poor differentiation states with features favoring rapid growth^52,53^. Interestingly, as shown in Fig. 6d, BMP4 expression levels inversely correlated with the increasing tumor grade. To further explore the relationship between BMP4 gene expression and patient clinical outcomes, we also performed Kaplan–Meier analysis^54^, using a large cohort of 3557 breast cancer patients. As shown in Fig. 6e, low BMP4 expression significantly correlated with poor relapse-free survival, while TGFβ expression showed the opposite trend (Fig. 6f). The opposing clinical outcomes for BMP4 and TGFβ are consistent with our findings, whereby expression of pro-differentiation factors, such as BMP4 efficiently reduces CSC stemness and correlates with less aggressive tumors and much improved patient survival outcomes, opposite to what observed with stemness factors, such as TGFβ.

## Discussion

Cancer stem cells are emerging as an attractive clinical therapeutic target for many types of cancer. In breast cancer, many reports have indicated that BCSCs are associated with resistance to conventional therapies such as chemotherapy or radiotherapy, and have the ability to regrow tumors resulting in later relapse of breast cancer patients^55,56^. In particular, the TNBC molecular subtype is highly enriched in cancer stem cells and exhibits a high incidence of distant relapse disease following chemotherapy treatment^3^. To date, there is no efficient targeted therapy for this type of cancer, thus defining a clear unmet medical need for these TNBC patients. As such, a better understanding of the molecular mechanisms underlying the regulation of stem-like properties of BCSCs and identification of the upstream growth factor signaling pathways that control these events will be instrumental for the development of novel clinical therapeutic strategies against TNBC.

Components of the TGFβ signaling cascade, including its receptors and downstream target genes, are highly expressed in ER- breast tumors, enriched in CD44^+^/CD24^−/low^ cancer stem cells, and their expression is associated with a significant shortening of distant metastasis-free survival outcome^23^. In this study, we found that TGFβ significantly promotes the self-renewal activity of cancer stem cells in TNBC and that blocking TGFβ type I receptor kinase activity with a specific small-molecule inhibitor efficiently prevented these effects. These results indicate that TGFβ signaling plays a prominent role in perpetuating stemness in breast cancer, and are in line with the previously established pro-migratory/invasive/metastatic role exerted by this growth factor in advanced, aggressive TNBC tumors^37,57–60^. Thus, targeting specific components of the TGFβ signaling pathways represents an interesting option for efficiently targeting cancer stem cells and for treating TNBC patients with recurrent locoregional or metastatic tumors.

Cyclin D1 is one of the critical regulators of embryonic, hematopoietic, and mammary stem cells^29,61–63^. Deregulation of cyclin D1 expression has been observed in many types of human cancers^64^. A correlation between overexpression of cyclin D1 and poor clinical outcomes has been also been established^65,66^. We previously showed that cyclin D1 cooperates with p21 to regulate TGFβ-mediated breast cancer cell migration and tumor local invasion through transcriptional regulation of Smad activity in a CDK4-independent manner^37^. We showed here that cyclin D1 is required for TGFβ-mediated stem cell activity and self-renewal in TNBC cells. Interestingly, cyclin D1 was previously found to be required for the self-renewal of mammary stem and progenitor cells that are targets of MMTV-ErbB2 tumorigenesis^63^. Thus, cyclin D1 may play a broader role in regulating the activity and self-renewal properties of various progenitor cells in various breast tumors of different molecular subtypes. Our results also strengthen previous findings highlighting cyclin D1 as an important therapeutic target in cancer^67^.

Within the TGFβ superfamily, the TGFβs maintain embryonic stem cell pluripotency and self-renewal capacity by modulating gene expression of pluripotent transcriptional factors (Nanog, Oct4, Sox2), while other members, such as the BMPs, act as embryonic stem cell differentiation factors^68,69^. In cancer, BMP4 was shown to promote CSC differentiation, leading to diminished tumorigenic capacity and increased sensitivity to chemotherapy drugs in hepatocellular carcinoma and colorectal cancer models^70,71^. However, BMP4 role and contribution to tumorigenesis remain controversial as some studies also suggested that BMP4 could exert a dual role and exhibit pro-migratory and pro-invasive functions in breast cancer^72,73^. We show here that BMP4 acts as a potent differentiation factor and prevents cancer stemness by inhibiting tumorsphere formation and reducing CD44^+^/CD24^−^ CSC numbers in TNBC. Consistent with this, we found that BMP4 expression is lower in basal-like, ER− and high-grade breast tumors, all of which being enriched in BCSC and having the worst prognostic features. Considering the difference in CSC content observed between the different molecular breast cancer subtypes^74^, this suggests that BMP4-targeting therapies should be primarily developed and be more efficient for CSC enriched/driven tumors, such as basal-like or TNBC. Finally, using normal mammary epithelial 3D cell culture assay, we also showed that BMP4 acts as a differentiation factor in normal cells and can induce the formation of 3D acinar structures, further broadening its role as a differentiation factor in normal and cancer cells. These effects of BMP4 on mammary acini morphogenesis, suppression of breast cancer stemness, and association of its expression with differentiated low-grade breast cancer subtypes are reminiscent of another key mammary differentiation factor, the prolactin hormone. Indeed, prolactin and its receptor were also shown to mediate mammary acini morphogenesis^48^ and their expression was also observed to correlate with less aggressive breast cancer phenotypes, including low-grade tumors and luminal breast cancer subtype^75,76^. Interestingly, we also previously found antagonistic cross-talk between TGFβ and prolactin in breast cancer^77^. Altogether, these findings provide evidence supporting the notion that mammary differentiation factors may provide opportunities for the development of much needed cancer stem cells targeted therapeutics.

**Fig. 7:**
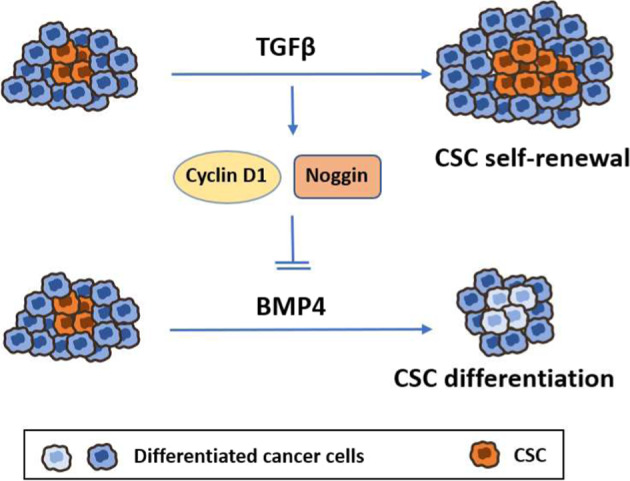
Novel BMP4/TGFβ interplay in the regulation of cancer stemness. Graphical representation of the TGFB, BMP4, and noggin feedback-loop mechanism in regulating cancer stem cell self-renewal activity in TNBC.

In summary, we defined a novel interplay between TGFβ family members in the regulation of cancer stemness. As represented in Fig. 7, we showed that TGFβ could act in a powerful feedback loop to repress BMP4 expression while inducing expression of the BMP4 inhibitor, Noggin, and as a result promote CSC self-renewal in TNBC. We further found TGFβ and BMP4 to antagonize each other effect on cancer stemness in high-grade, invasive basal-like tumors, and show that their relative expression (high TGFβ/low BMP4 levels) correlated with poor prognosis and survival outcomes. This study opens up new avenues for developing anti-CSC therapies targeting TGFβ signaling (i.e., small kinase inhibitors) and/or using BMP4 mimics that could prove efficient as novel targeted therapies for TNBC patients.

## Supplementary information

Supplementary Material and Methods

## Data Availability

All data generated or analyzed during this study are included in this published article and its Supplementary Information files.
