# Supplementary material for: TGFβ/cyclin D1/Smad-mediated inhibition of BMP4 promotes breast cancer stem cell self-renewal activity
Source: Oncogenesis. 2021 Mar 1;10(3):21. doi: 10.1038/s41389-021-00310-5 (PMC7921419; doi:10.1038/s41389-021-00310-5)
Supplement: Supplementary file 1 — Supplementary Material and Methods [file 41389_2021_310_MOESM1_ESM.docx]

**Cell transfection:** 30 nM scramble (control), cyclin D1 siRNAs (Sigma) was transfected into SUM159PT or SCP2 cells using Lipofectamine^TM^ 2000 (Invitrogen, Carlsbad, CA, USA) for overnight in serum-free medium, according to the manufacturer’s protocol. Post-transfection 24 hours, cells were then treated with 100 pM TGFβ1 (Peprotech Cat#100-21).

15 µg shRNAs targeting scramble(control), Smad2, Smad3, Smad4 (sigma), and packaging plasmids of 12 µg psPAX2 (Addgene, 12260) and 4.5 µg pMD2.G (Addgene, 12259) were transfected into HEK293T cell using 80 µl of 1 mg/ml polyethylenimine PEI (Sigma) for overnight in a 10 cm plate. psPAX2 and pMD2.G were a gift from Didier Trono. The medium was then changed with fresh 10 ml DMEM with 10% FBS. ShRNA lentiviruses were collected from cell supernatants after 24 hours. SUM159PT and SCP2 cells were infected with shRNA lentiviruses with 8µg/ml polybrene for overnight. Post-infection 36 hours, cells were then selected by puromycin for 2 days.

**Tumorsphere formation assay**: Tumorspheres were imaged by microscopy. Tumorsphere-forming efficiency was calculated as the number of mammospheres divided by the number of singles cells seeded, expressed as a percentage. Where indicated, the TGFβ1 and BMP4 (Peprotech Cat#120-05) recombinant ligands and 10 µM TGFβ type I receptor (TβRI) inhibitor (SB431542, Sigma, cat#S4317) were added at the final concentration presented in the figures based on experimental design.

**Flow cytometry analysis:** Monolayer cells were dissociated into single cells and filtered through a 40 µm cell strainer. 500,000 cells were incubated in prechilled PBS with 2% FBS for half an hour at 4 ℃.Samples were further incubated with anti-CD44 conjugated to APC (APC mouse anti-human CD44, BD Bioscience Cat#559942), anti-CD24 conjugated to PE (PE mouse anti-human CD24, BD Bioscience Cat#555428) for 30 minutes. Isotype-matched conjugated non-immune antibodies were used as negative controls. Cells were then washed 3 times with FACS buffer and analyzed with Accuri C6 flow cytometer (BD Biosciences) and Flowjo software (Tree Star Inc.).

**Real time PCR:** Random hexamers and M-MLV Reversed Transcriptase (Invitrogen) were used in the reverse transcription. The real-time qPCR was performed with SsoFastTM EvaGreen® Supermix (Bio-Rad) using a RotorGene 6000 PCR thermocycler. The RT-qPCR steps are: 95 °C for 30 s, 40 cycles of 95 °C for 5 s, and 60 °C for 20 s. The primers of BMP4 and Noggin were used to quantify their mRNA expression levels.

**Western blot analysis:** Monolayer or mammosphere cells lysis were extracted using chilled RIPA buffer containing 10 mm Tris-HCl, pH 7.5, 5 mm EDTA, 150 mm NaCl, 30 mm sodium pyrophosphate, 50 mm sodium fluoride, 1 mm sodium orthovanadate, 1% Triton X-100 and protease inhibitors (1 mm phenylmethylsulfonyl fluoride, 10 µg/ml leupeptin hydrochloride, 10 µg/ml aprotinin and 10 µg/ml pepstatin A) at 4°C. Total protein concentration was quantified using a BCA protein assay kit (Thermo Scientific, Cat#23227). Cell lysate samples were incubated in 6×sodium dodecyl sulfate (SDS) buffer for at 95 ℃ for five minutes and immunoblot analysis was performed using antibodies against Smad2/3 (Santa Cruz Biotechnology, Cat#sc-6032), Smad4 (Santa Cruz Biotechnology, Cat#sc-7966), cyclin D1 (thermo scientific Cat. #MS-210-P0). The anti-beta Tubulin antibody (Santa Cruz Biotechnology, Cat#sc-5274) as loading control.

**Luciferase assay:** Cells were transfected with 0.5 µg individual BMP4 promoter plasmid and 0.1 µg pCMV-β-GAL for overnight. Post-transfection 24 hours, cells were then treated with 100 pM TGFβ1 for 24 hours. Cell samples were lysed by extraction buffer containing 1% Triton X-100, 15 mM MgSO_4_, 4 mM EGTA, 1 mM DTT and 25 mM glycylglycine. Cell lysates were mixed with a cocktail containing 0.1 M ATP, 0.5 M KH_2_PO_4_ and 1 M MgCl_2_. Luciferase activity was quantified using luminometer and normalized to β-galactosidase activity.

**3D cell culture:** The Poly-D-Lysine coated 8-well culture slides (BD Biosciences) were used for 3D culture. Concisely, each well of the culture slide was coated with 100 µl growth factor reduced Matrigel® (BD Biosciences). 4,000 cells were plated in each well. Cells were grown and maintained in RPMI growth medium with 5% Matrigel® for 48 hours. The morphology of mammary epithelial organoids was evaluated after 72 hours of different treatments: (1) control (Ctrl): 2% FBS, (2) TGFβ: TGFβ 100 pM and 2% FBS, (3) BMP4: BMP4 100 ng/ml and 2% FBS or (4) BMP4/TGFβ: BMP4 100 ng/ml , TGFβ 100 pM and 2% FBS. Mouse primary MECs were isolated and prepared from virgin C57BL/7 (Jackson Mice) females in RPMI media with 10% FBS using a kit, STEMCELL Technologies INc. (Canada).

**Gene expression profiling:** All procedures for RNA purification, RNA quality control and concentration determination were performed at McGill University and Genome Quebec. RNA samples were amplified, labeled and further hybridized on Illumina HumanHT-12 v3 Expression BeadChip microarrays according to the manufacture’s protocol. The raw data were obtained by preprocessing the image data with Illumina software. The raw data were normalized and further analyzed for differential gene expression profiling using Limma package (version 3.44).

**Immunofluorescence staining and confocal microscopy:** mammary organoids in 3D culture were fixed in 4% PFA for 1 hour at room temperature. Organoids then were permeabilized in 0.5% Triton X-100/1XPBS (PBST) for 5 minutes and blocked with 5% normal donkey serum in 0.5% PBST for 1 hour. Organoids were subsequently immunostained with primary antibodies of anti-E-Cadherin rat monoclonal antibody (Sigma #U3254), anti-ZO1 mouse monoclonal antibody Alexa Fluor® 488 (Introgen #339188) for an overnight period at 4ºC followed by secondary antibody of goat anti-rat IgG (H+L) Fluor 555 (Invitrogen #A21434) and DAPI for 1 hour at room temperature. Samples were mounted in FluorSaveTM (CALBIOCHEM®). Samples were imaged on a Zeiss 510 or 780 LSM confocal microscope with an Axivert 200M microscope and a C-Apochromat 63x/1.2W Core lens.
